# Overcoming subject variability in P300 decoding based on quantification by deep learning

**DOI:** 10.3389/fnins.2026.1940267

**Published:** 2026-10-22

**Authors:** Maohua Liu, Fred R. Beyette

**Affiliations:** School of Electrical and Computer Engineering, University of Georgia, Athens, GA, United States

**Keywords:** deep learning, MoE, P300 decoding, quantification, subject variability

## Abstract

Binary P300 decoding is a cornerstone of P300-based brain-computer interfaces, enabling applications such as assistive communication, neurorehabilitation, and cognitive monitoring. However, substantial subject variability in EEG patterns poses a major challenge to the development of generalized P300 decoders. To address this problem, this study proposes SQ_EMoE, a subject-variability-aware enhanced Mixture-of-Experts framework that uses the confidence produced by a deep subject-identification network as a practical proxy for trial-level subject distinctiveness. High-confidence trials are routed to subject-specific experts, whereas uncertain trials are redirected to a pooled general expert, allowing the framework to dynamically balance subject-specific specialization and cross-subject generalization. Latent subject representations are further visualized to qualitatively examine subject separability and its relationship with routing behavior. Experiments on eight random subjects from each of two public P300 datasets yielded mean ROC–AUC values of approximately 0.86 on BNCI Horizon 2020 and 0.89 on P300-LINI, outperforming pooled EEGNet and architecture-matched pooled P300MCNN baselines. Ablation experiments further demonstrated the contribution of confidence-controlled fallback to the general expert. To evaluate scalability, the framework was additionally tested on all 22 subjects of P300-LINI. With the original subject-identification network, SQ_EMoE achieved a mean ROC–AUC of 0.8518; after strengthening the subject representation and identification network, performance increased to 0.8760, exceeding the pooled P300MCNN baseline (0.8482) and slightly surpassing the oracle routing or within-subject decoding (0.8744). Leave-one-subject-out experiments further showed that the under-realized SQ_EMoE conservatively redirects most completely unseen-subject trials to the general expert when reliable subject-specific routing is unavailable. These results demonstrate that effective modeling of subject variability, together with adaptive integration of shared and subject-specific representations, provides a promising alternative to conventional one-size-fits-all EEG decoding.

## 1 Introduction

Binary P300 decoding is a cornerstone for brain-computer interfaces (BCIs), enabling reliable communication, neurorehabilitation, and cognitive monitoring for differently abled individuals. As a prominent event-related potential (ERP) appearing ~300 ms post-stimulus (Wolpaw, 2007), the P300 wave powers stimulus-driven, dependent BCIs. Compared to self-initiated BCIs, these systems require less cognitive effort and offer greater robustness for patients with neurological disorders (Farwell and Donchin, 1988).

Despite its clinical promise, binary P300 decoding is severely challenged by substantial subject variability in EEG patterns (Treder and Blankertz, 2010). This physiological heterogeneity dramatically degrades the generalizability of standard decoders, rendering cross-subject decoding highly inaccurate for unseen users (Lotte et al., 2018). Consequently, conventional systems suffer from a severe calibration dependency, requiring time-consuming and tedious subject-specific calibration prior to practical deployment (Sellers et al., 2010).

This variability arises from individual differences in brain anatomy, neurophysiological responses, cognitive profiles, attention, fatigue, and learning curves (Zander et al., 2010; Blankertz et al., 2011). Consequently, the amplitude, latency, and morphology of the P300 wave vary widely across populations, making a universal decoding model exceptionally challenging (Fazel-Rezai et al., 2012; Hajinoroozi et al., 2016). A decoder optimized for one individual often performs poorly on another, necessitating frequent recalibration. Current approaches to mitigating this variability generally fall into three categories.

The first category holds that subject variability is not so significant as to be irreconcilable by a model with broad generalization capabilities; consequently, this approach focuses on continuously improving the model’s generalization capability. Over the past decade, scaling parameters, engineering complex architectures, and expanding datasets have yielded suboptimal results in reconciling subject variability. Early attempts focused on stacking convolutional neural network (CNN) layers, peaking with CNN-R at ~20 million parameters (Cecotti and Graser, 2010; Manor and Geva, 2015). However, a paradigm shift toward lightweight models began with DeepConvNet (Lotte et al., 2018) and EEGNet, which reduced parameters to around 1,000 using depth-wise convolutions while achieving state-of-the-art cross-subject decoding (Lawhern et al., 2018). Recent iterations like P300MCNN have pushed miniaturization to just 161 parameters without sacrificing performance (Alvarado-Gonzalez et al., 2021; Liu et al., 2023; Liu et al., 2024). This lightweight trend revealed that the strong inductive bias of CNNs limits their generalization to heterogeneous EEG data, prompting researchers to explore weak inductive bias architectures like the Transformer (Kuruppu et al., 2026). Inspired by the predictable scaling laws and foundational success of Transformers in NLP and CV (Vaswani et al., 2017; Hoffmann et al., 2022; Zhai et al., 2022), researchers developed massive EEG foundation models like BENDR (Kostas et al., 2021), BIOT (Yang et al., 2023), and LaBraM (Jiang W. -B. et al., 2024) via self-supervised pre-training. However, brain decoding has not mirrored the scaling success of NLP and CV. Because EEG data lacks universal semantic foundations and remains highly heterogeneous, brute-force scaling yields severely diminishing returns (Yang et al., 2023, 2026; Jiang W. -B. et al., 2024).

The second category posits that subject variability exceeds the generalization capacity of a single model, necessitating Mixture-of-Experts (MoE) architectures (Jiang A. Q. et al., 2024; Riquelme et al., 2021). MoE models decompose computation across multiple expert networks using a gating mechanism to route inputs (Jacobs et al., 1991), while sparsely gated variants scale model capacity without proportional computational costs (Shazeer et al., 2017; Fedus et al., 2022). Recently, MoE has been adapted for brain decoding: EEGMamba (Gui et al., 2024) pairs a task-aware learned gating network with a universal expert to capture shared EEG structures, whereas MoGE (Liu et al., 2024) and VMoGE (Ding et al., 2025) utilize graph-based experts assigned to specific channels or frequency bands. In this context, subject identity serves as a deterministic, fixed gating signal that routes inputs directly to individual expert pathways.

The third category occupies a middle ground, positing that EEG representations comprise both transferable, subject-agnostic components and subject-specific variations. This is supported by scaling law analysis showing that neural decoding performance improves with more training data per subject, but scales poorly with the number of subjects (Banville et al., 2025). Cross-subject benchmarks (Wang et al., 2020; Kwon et al., 2020) demonstrate that cross-subject transfer is generally possible, although with notable performance degradation. Taken together, these findings suggest that brain decoding representations comprise both transferable subject-agnostic components and subject-specific components that limit performance gains when scaling across subjects. This category currently remains primarily at the conceptual stage. Methods have emerged that employ a shared model to process subject-agnostic components while using independent structures to handle subject-specific components separately; results indicate that this approach yields superior outcomes (Klein et al., 2025). However, these methods merely assume the simultaneous presence and need for separate processing of subject-agnostic and subject-specific components, without quantifying the extent of either or implementing targeted processing strategies based on their respective proportions.

As shown in Figure 1, the three categories described above are suited to situations with different level of subject variability. The first applies to situations where subject variability is small, as shown in Figure 1a; in such cases, a single, unified model can be used to accommodate these variations, and the smaller the difference in subject variability, the more effective this category is. The second category applies to situations with significant subject variability, requiring multiple model architectures (such as MoE) to handle distinct subject distributions separately, as shown in Figure 1b; the greater the subject variability, the more effective this category becomes. The third category falls between the first two and requires a more flexible approach. The key to determining which specific situation applies lies in the level of subject variability; therefore, quantifying the level of subject variability is unavoidable so as to select the most suitable solution for different situations. This approach essentially abandons the “one-size-fits-all” model strategy. Instead, it employs a set of lightweight models that can be updated as needed, ensuring that every subject is matched with the model best suited to them. The extreme lightweight nature of the models used for P300 decoding has transformed this strategy from one that was never considered into a feasible option. This strategy is applicable to a wide range of scenarios. Consider, for instance, a TV remote control operated via EEG-based BCI. Such a remote might be used by no more than thirty people over its entire lifespan—and by ten or fewer individuals for the vast majority of the time. By preparing a lightweight model for each of these ten users and selecting the appropriate one dynamically, the need for calibration can be almost entirely eliminated. Unlike methods involving repetitive, day-to-day calibration without saving the results, ultra-lightweight models can store the outcome of each calibration in just a few hundred bytes; they maintain and update a set of frequently used weights—functioning much like a cache or system memory—enabling users to obtain satisfactory weights through direct selection rather than recalibration.

**Figure 1:**
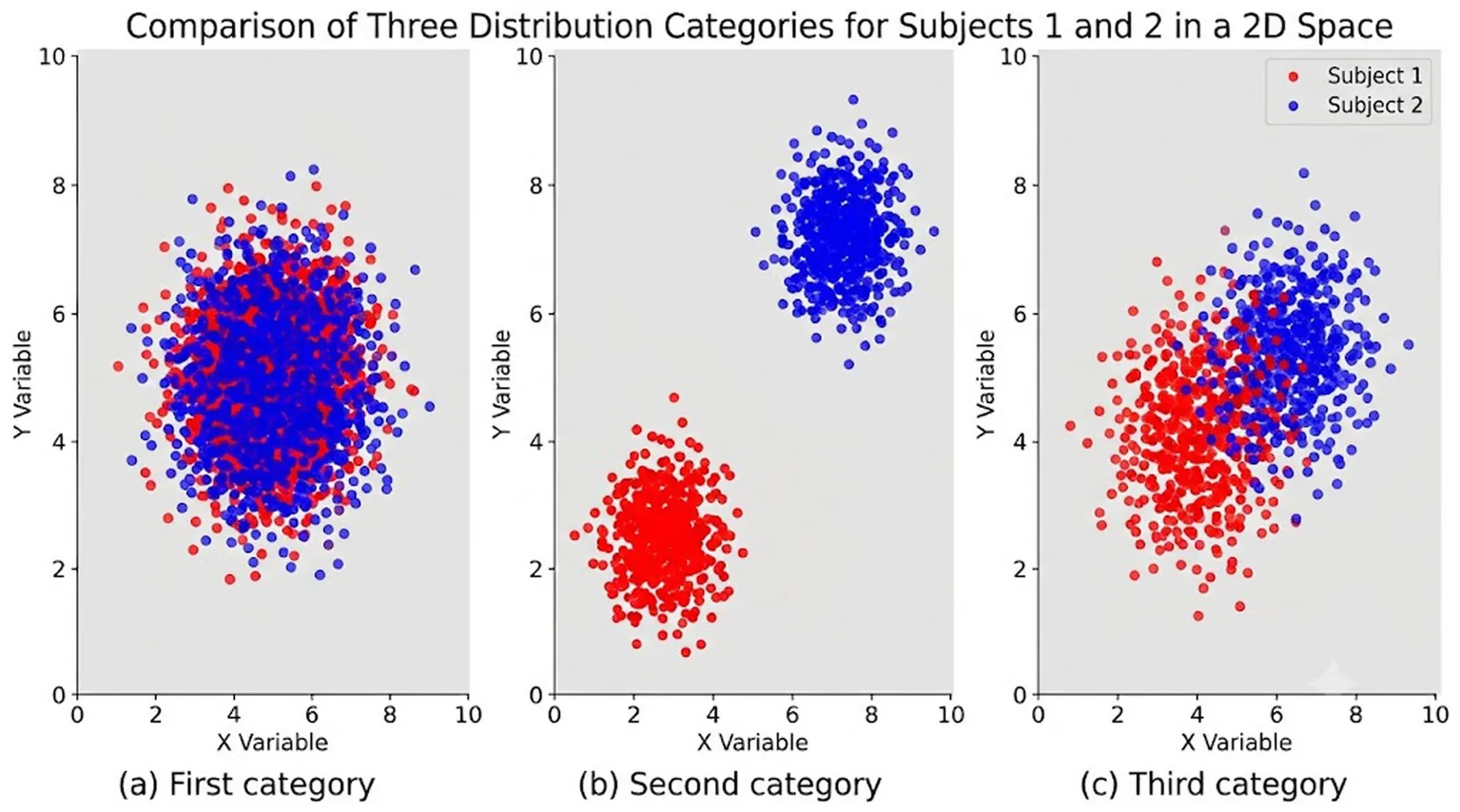
Illustration of three categories of subject variability. **(a)** First category: low subject variability, characterized by substantial overlap between the data distributions of different subjects. **(b)** Second category: high subject variability, characterized by clearly separated subject distributions. **(c)** Third category: intermediate subject variability, characterized by partially overlapping subject distributions. Red and blue points represent data from two different subjects.

This study aims to develop a method applicable to all situations based on the quantification of subject variability. The main contributions of this study are as follows: First, it makes the first attempt to quantify the magnitude of subject variability with deep learning, specifically leveraging the confidence level of deep learning network on subject identification. Second, it proposes an enhanced MoE framework capable of handling varying degrees of subject variability accordingly. Third, the proposed method is also widely applicable to other data exhibiting significant subject variability.

## 2 Methods

The extent of subject variability is not fixed but varies depending on the context. Assuming a fixed context and applying any one of the three aforementioned categories to handle all others would yield poor results. This study aims to establish a method applicable to any category or context. This method is named SQ_EMoE because it primarily consists of two modules: subject variability quantification, and enhanced MoE. The following sections detail SQ_EMoE’s framework, its two important modules, and the experimental setup.

### 2.1 Framework

As shown in Figure 2, the method proposed in this study integrates processing approaches for the first and second categories and is also applicable to the third category. For each data point, the subject to which it belongs is first identified. The subject identification network outputs confidence probabilities regarding the data’s association with each subject to the subsequent quantization and routing unit. This unit calibrates the classification result based on the confidence probabilities output by the subject identification network. If the confidence level is higher than the threshold, the classification result is maintained; if the confidence level is lower than the threshold, the data is routed to a general-purpose expert rather than to the expert associated with the subject selected by the subject identification network. For instance, if subject variability is small, confidence levels tend to be low due to the difficulty of identification, resulting in the data mostly being assigned to the general-purpose model—a scenario corresponding to the first category. Conversely, if subject variability is substantial, confidence levels rise, leading to the data mostly being assigned to specific sub-experts—a scenario corresponding to the second category. Finally, if subject variability falls somewhere in between, the system can flexibly adapt to the situation by adjusting the confidence threshold. The following is an introduction to each of the main modules.

**Figure 2:**
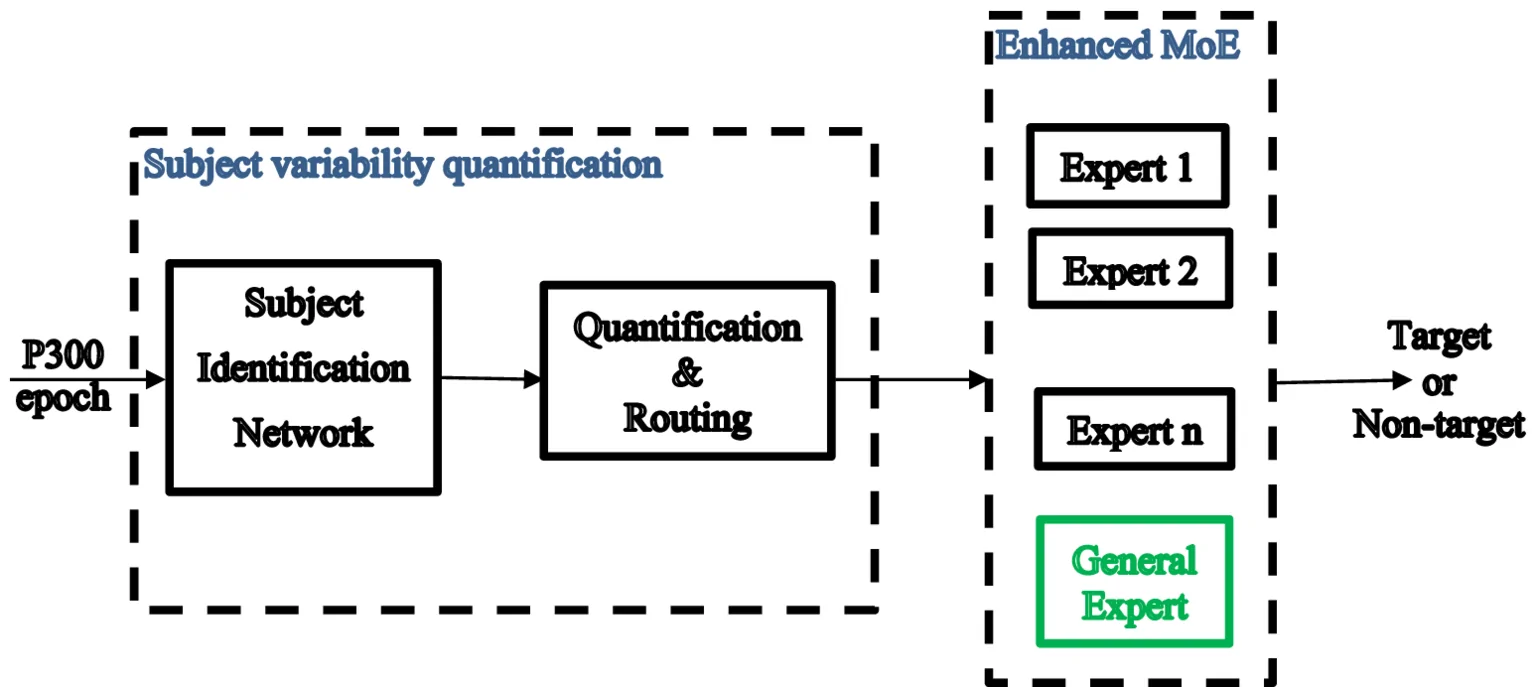
The framework of SQ_EMoE.

### 2.2 Subject variability quantification

As mentioned in the introduction section, subject variability refers to the distinct differences in brainwave patterns across individuals—that degrade the cross-subject generalization performance of machine learning models. Subject variability can be defined as the average distance $D_{\mathrm{avg}}$ between the distributions of any two subjects, as the show in Equation 1:

$$\text{subject variability}=D_{\mathrm{avg}}=\frac{2}{N(N-1)}\sum_{i=1}^{N}\sum_{j=i+1}^{N}\;d(P_{i},P_{j})$$ (1)

*N* represents the total number of subjects; $P_{i}$ and $P_{j}$ denote the data or feature distributions of subject *i* and subject *j*. Applying Equation 1 to the three cases in Figure 1 reveals that Figure 1a exhibits low subject variability ( $d(P_{i},P_{j})\approx 0$ ), Figure 1b is high, and Figure 1c falls in between.

While it is easy to provide definitions or formulas, performing calculations based on Equation 1 using real-world data is difficult because it involves distributions. Distributions—particularly high-dimensional ones—are challenging to parameterize and quantify. Fortunately, deep learning can make up this gap. Deep learning is a black box which can approximate any function; therefore, it can be used to approximate the distances of any two distributions. In order to train the deep learning model to approximate the distribution distances, you must find an appropriate task for it. We find that subject identification is the perfect choice, which means, the quantification of subject variability can be implemented based on the subject identification task, as there is an inherent link between the two. High subject variability results in high identification accuracy, whereas low subject variability leads to poor identification accuracy, the two are proportional, as shown in Equation 2. To capture the idiosyncratic identity traits embedded within EEG signals, we implement a specialized convolutional neural network structured on the principle of spatial–temporal decoupling. The network processes multi-channel EEG trials through a sequential feature-extraction strategy that segregates the learning of temporal dynamics from spatial topography. Initially, a temporal convolution layer operates along the time axis for each channel independently, serving as a frequency filter bank to capture transient neural dynamics. Following this, a depth-wise convolutional layer spans across the entirety of the electrode channels, acting as a data-driven spatial filter to combine distinct channel topologies into person-specific spatial patterns. To refine these integrated features while maintaining parameter efficiency, the network subsequently utilizes depth-wise separable convolutions, which heavily curtails the parameter footprint and prevents overfitting to noisy EEG backgrounds. After feature extraction, the outputs are flattened and projected into a dense 64-dimensional latent space, designated as the subject embedding layer, which serves as a highly discriminative “neural fingerprint” of the participant. Finally, the identity output layer utilizes a Softmax activation function to map these embeddings into a probability distribution over the *N* candidate identities, where each value represents the confidence level of the input EEG trial belonging to a specific subject. If the confidence level is high for the identification result of one data, that means, regarding the attribution of this data, the boundaries between individual subjects are distinct and the scope for dispute is minimal, yet there is significant difference among the subject distributions. On the contrary, if the confidence level is low for the identification of one data, that means, it is difficult to decide which subject this data belong to, indicating a low subject variability. Consequently, the confidence level of the deep learning network can serve as a proxy for the magnitude of subject variability. Although conventional statistical methods exist for quantifying discrepancies between data distributions, these methods are generally ineffective for high-dimensional real data. Real-world data is complex and high-dimensional; thus, calculating the distribution parameters of real-world data using mathematical or statistical methods is virtually ineffective. Deep learning is a more realistic approach in this regard based on our experiments.

Suppose there are *N* subjects; the subject identification deep network outputs *N* probability values, *P*_1_, *P*_2_, … *P_N_*, each representing the confidence level that the input data belongs to a specific subject—effectively quantifying the perceived distinctiveness of that subject. A higher confidence level indicates that the subject is distinct from the others and easily identifiable, whereas confidence levels tending toward uniformity suggest that the subjects are similar and difficult to distinguish. In cases where differentiation is difficult, assigning the data to a general model is a safer strategy than assigning it to a specific subject with low confidence; the former resembles a random-guess paradigm, while the latter offers greater stability. Equation 2 illustrates the proportional relation between subject variability and the confidence level, thereby assist in routing to different experts based on the confidence level threshold defined by Inequality (3):

$$\text{subject variability}\propto \max(P_{1},P_{2},\ldots ,P_{N})$$ (2)

$$\max(P_{1},P_{2},\ldots ,P_{N})>\frac{1}{N}+0.1$$ (3)

If the output of the deep learning network satisfies Inequality Equation 3, it indicates high confidence, and the original decision is maintained; if the output does not satisfy Inequality Equation 3, it indicates low confidence—suggesting potential indecision or a stochastic uncertainty—in which case the data is reassigned to the general model for processing to avoid instability. In practice, this threshold can be adjusted to strike a balance between performance and stability. Particularly when finding a matching expert is difficult, this threshold should be set significantly higher. Note that there is no calibration used throughout the design and experiment and Equation 2 does not directly use confidence to represent subject variability. Subject variability is formally defined in Equation 1, which accounts for multiple factors, such as the class count (*N*). Because Equation 1 is difficult to compute directly in practice, deep learning and Equation 2 are used to estimate its underlying trend—hence the use of a proportional sign ( ∝ ) rather than an equal sign (=). Under consistent conditions, such as fixed task difficulty and noise levels, the confidence score provides a relatively fair metric for evaluating the change of subject variability, therefore supporting SQ_EMoE. Deep learning networks can also automatically filter out noise and capture the core features; this is corroborated by the visualization of the deep representation layers in the experimental results, where data from the same subject largely cluster together, while there are clear separations between data from different subjects.

### 2.3 Enhanced MoE

This enhanced MoE is fundamentally an MoE architecture comprising multiple expert models that cater to various data types, thereby improving decoding performance. The key distinction is the inclusion of a “generalist expert” designed to handle data with low classification confidence—specifically, data that is difficult to assign to a specific subject. All expert models, including the generalist expert, share the same architecture and are implemented using P300MCNN (Liu et al., 2024)—currently one of the most lightweight and high-performing P300 decoding models, with a parameter count of only 161. Consequently, the system can readily accommodate a significantly larger number of expert models without issue. Fundamentally, SQ_EMoE departs from the conventional paradigm of relying on a single model’s generalizability or scalability; instead, it is anchored in a space-for-time philosophy that replaces a traditional large model with a multitude of micro-models (Klein et al., 2025). For instance, the number of expert models can be 100,000 or even more. We hypothesize that a larger number of experts generally leads to better performance, as a vaster pool of candidates could make it easier to identify the expert best suited to the current input data and potentially eliminate the need for explicit calibration. Under such a massive expert configuration, it can also be hypothesized that traditional Leave-One-Subject-Out (LOSO) evaluation might theoretically no longer apply in the same way. However, these possibilities have not been demonstrated in the present study and remain important directions for future evaluation. Instead, in this study, we employed only eight expert models rather than a larger pool—leaving SQ_EMoE unconfigured to its full capacity. Despite this limited configuration, SQ_EMoE still achieved modest superior performance on the LOSO test by leveraging high confidence level thresholds.

### 2.4 Datasets

This study uses two datasets, the BNCI Horizon 2020 Dataset (Dataset 8) (Liu et al., 2026; Riccio et al., 2013) and the P300-LINI Dataset (Brunner and Müller-Putz, 2014), which are introduced below.

BNCI Horizon 2020 dataset is composed of EEG records from eight subjects with Amyotrophic Lateral Sclerosis (ALS), recorded by an 8-channel placement (Fz, Cz, Pz, Oz, P4, P3, PO8, and PO7) according to international 10–10 system. The right earlobe and left mastoid served as reference and ground, respectively. Signals were sampled at 256 Hz and bandpass-filtered from 0.1 to 30 Hz. The P300 speller matrix adopted an oddball paradigm, in which rows and columns flashed randomly 10 times at 4 Hz (125 ms flash duration, 125 ms inter-stimulus interval). Post-stimulus epochs of 800 ms were extracted, obtaining 700 target (P300) and 3,500 non-target trials for each subject.

P300-LINI contains EEG signals from 22 healthy student subjects (aged 21–25). Although recorded with 10 channels under international 10–20 system (right earlobe reference, right mastoid ground), an optimized 6-channel subset (Fz, Cz, Pz, PO8, Oz, and PO7) was selected due to high relevance for P300 detection (Ledesma-Ramirez et al., 2010; Alvarado-González et al., 2016). Signals were sampled at 256 Hz and online-filtered by a 4th-order notch filter (58–62 Hz) and 8th-order Chebyshev bandpass filter (0.1–60 Hz). The oddball paradigm flashed matrix rows and columns every 125 ms (62.5 ms duration, 15 iterations per trial), yielding 480 target (P300) and 2,400 non-target epochs per subject. Offline preprocessing extracted 800 ms post-stimulus epochs, applied a 4th-order Butterworth bandpass filter (0.1–12 Hz) to isolate ERPs, subtracted the mean for DC offset removal, and performed linear detrending.

### 2.5 Experiment setup

All preprocessing, model training, and evaluation pipelines were implemented based on the open-source TensorFlow/Keras framework and standard scientific libraries, including NumPy, SciPy, and Scikit-learn. To achieve high computing speed and efficient model acceleration, all experiments were conducted on the cloud-based Google Colab Enterprise platform. The hardware environment utilized an enterprise-grade NVIDIA A100 Tensor Core GPU (with 40 GB/80 GB HBM2e memory) together with a high-RAM virtual machine configuration. Under this hardware setup, parallel processing for 5-fold cross-validation iterations was accelerated, and gradient calculation during joint training of the subject identification network and MoE modules was expedited.

A key part of the experiment was to record the dynamic expert selection in the SQ_EMoE framework. We recorded the explicit gating routing decisions (i.e., routing paths assigned to each specific expert) and classification confidence levels during cross-validation. To test whether the proposed subject identification stage can effectively quantify and capture individual neural differences, latent feature representations were extracted from network layers. These high-dimensional features were then mapped into a two-dimensional space using t-Distributed Stochastic Neighbor Embedding (t-SNE). This visualization provides an intuitive view of subject variability and cluster separation among different subjects.

Different baselines and ablation experiments are implemented. The architecture-matched and mechanism-focused comparisons are implemented. First, a pooled P300MCNN general model is trained on data from all training subjects and evaluated without routing. This baseline isolates whether the complete SQ_EMoE gains arise from expert routing rather than simply from the lightweight classifier backbone. Second, a pooled EEGNet model is evaluated under the same data partitions as an established EEG decoding baseline. Third, a conventional learned-gating MoE: every epoch is forced to the expert selected by the subject router regardless of confidence. Comparing this ablation with the complete SQ_EMoE directly assesses the contribution of confidence-based fallback to the general expert.

### 2.6 Leave-one-subject-out evaluation for unseen subjects

Besides of above baselines and ablation experiments, in order to test generalization to a user whose identity is absent during training, a LOSO protocol is implemented, and the performance of SQ_EMoE is compared with a pooled EENet baseline. In each LOSO round, one subject is completely excluded from model fitting and validation. The remaining *N −* 1 subjects provide the training data for the subject-specific experts, the pooled general expert, the subject-identification router, and the training-only channel scaler. The held-out subject is used only for final evaluation. Consequently, the LOSO experiment requires no subject-specific calibration samples from the test user.

Because the router has no output class corresponding to the unseen subject, its Softmax probabilities in LOSO represent similarity/confidence relative only to the known training subjects. The full LOSO SQ_EMoE therefore uses a conservative fallback threshold so that an unseen epoch is assigned to the pooled general expert unless the router is exceptionally confident threshold that it resembles one of the known subjects. Both the confidence threshold for the P300-LINI LOSO settings and for the Horizon LOSO settings are beyond 0.9. This protocol evaluates calibration-free decoding in the specific sense that no data from the held-out user are used for training, validation, normalization fitting, or expert construction; it does not assume that an unseen user can explicitly be identified.

### 2.7 Evaluation and diagnostic analyses

Binary P300 decoding is evaluated primarily with the ROC–AUC, which is appropriate for the class-imbalanced P300/non-target setting. The result reports subject-level ROC–AUC, fold-level/average ROC–AUC, routing counts, and the number of epochs sent to the general expert. For LOSO, where the test subject has no corresponding router class, the subject-level ROC–AUC, average ROC–AUC and general-expert fallback rate is recorded.

The subject-identification network’s 64-dimensional embedding can be projected with t-SNE for qualitative inspection of the learned representation. Such two-dimensional projections are treated only as diagnostic visualizations and not as direct evidence of physiological subject variability.

## 3 Results and discussion

The experimental evaluation was organized into two complementary settings. In the known-subject setting, each subject was represented during training and had a corresponding subject-specific expert, allowing us to evaluate the benefits of subject-aware routing and expert specialization. In the unseen-subject setting, a LOSO protocol was adopted, where the test subject was completely excluded from training and had no corresponding subject-specific expert. Both settings were evaluated on the BNCI Horizon 2020 Dataset and P300-LINI datasets to assess the effectiveness and generalizability of SQ_EMoE across different P300 datasets.

### 3.1 Known-subject evaluation

In the known-subject setting, each evaluated subject was represented during training and had a corresponding subject-specific expert. The application scenario addressed in this study dictates that the known-subject setting is the optimal evaluation method.

#### 3.1.1 BNCI horizon 2020

As shown in the right panel of Figure 3, the subject identification network achieved strong discrimination among the eight subjects. The two-dimensional t-SNE projection of the 64-dimensional subject embeddings shows eight largely distinct clusters, indicating that the network learned subject-discriminative representations from the EEG data. Nevertheless, some overlap and misclassification can be observed. In particular, some samples from Subject 2 were assigned to Subject 1, while some samples from Subject 3 were assigned to Subjects 2 and 7. These misclassifications help explain the high-confidence misrouting observed in the left panel of Figure 3, where some samples were routed to incorrect subject-specific experts despite high identification confidence.

**Figure 3:**
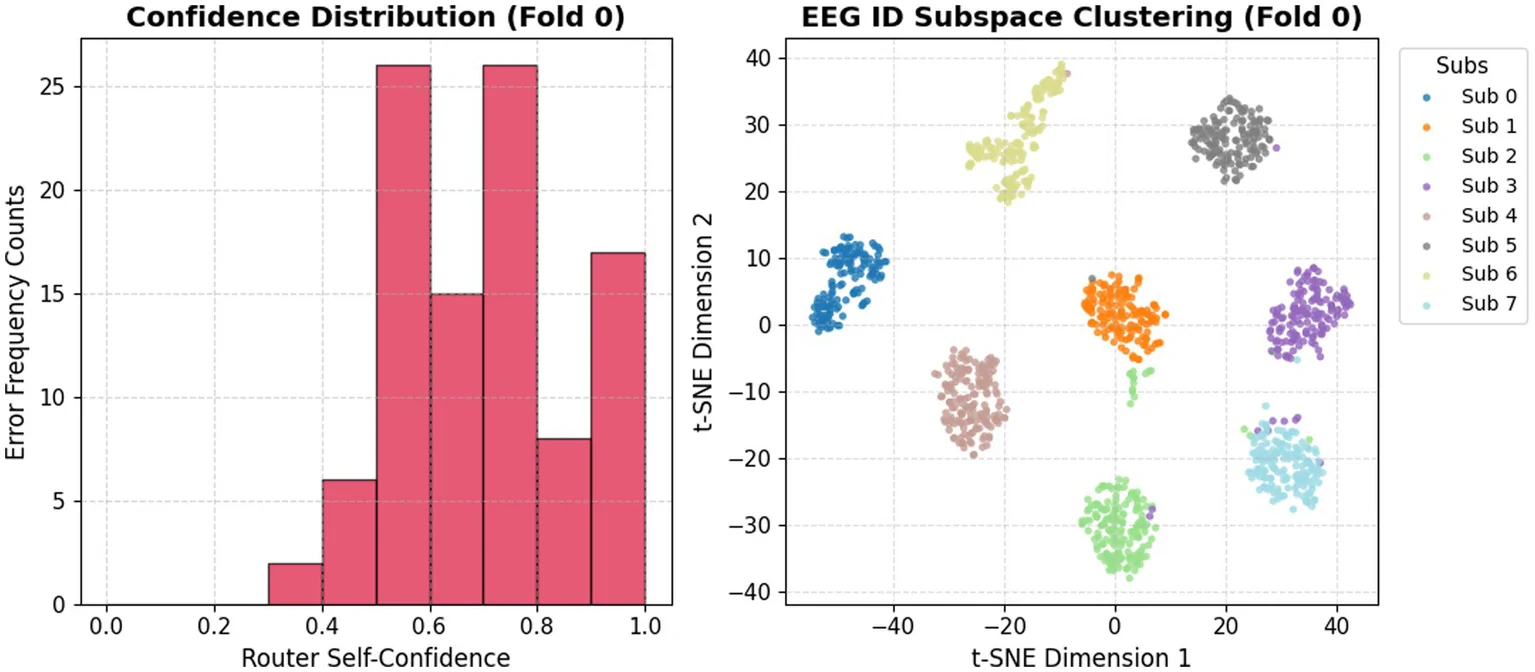
Subject identification and routing analysis for eight subjects from the BNCI Horizon 2020 dataset. (Left) Distribution of router confidence scores for incorrect routing instances. (Right) Two-dimensional t-SNE visualization of the 64-dimensional subject embeddings learned by the subject identification network.

Table 1 presents the subject-wise P300 decoding performance of the proposed SQ_EMoE framework on the BNCI Horizon dataset using all eight subjects. The results are reported in terms of ROC–AUC across five cross-validation folds. SQ_EMoE achieved an overall mean ROC–AUC of 0.8586, with the subject-level average ROC–AUC ranging from approximately 0.80 to 0.95. Despite substantial differences in decoding performance across individual subjects, the framework maintained relatively stable performance across the five folds, with fold-wise mean ROC–AUC values ranging from 0.84 to 0.87. These results demonstrate that SQ_EMoE can accommodate heterogeneous subject-specific decoding characteristics within the same multi-subject setting.

**Table 1:** Subject-wise P300 decoding performance of SQ_EMoE across all eight subjects.

Folds	Subjects								
	Subject 0	Subject 1	Subject 2	Subject 3	Subject 4	Subject 5	Subject 6	Subject 7	Subject mean
Fold 0	0.7711	0.8251	0.8848	0.8463	0.8605	0.8711	0.8764	0.9231	0.8573
Fold 1	0.8385	0.8315	0.8955	0.7929	0.8830	0.8766	0.8786	0.9620	0.8698
Fold 2	0.7946	0.8131	0.8733	0.7939	0.8674	0.8902	0.8622	0.9565	0.8564
Fold 3	0.7892	0.7333	0.8916	0.8284	0.8583	0.8408	0.8305	0.9506	0.8403
Fold 4	0.7856	0.8610	0.8863	0.7980	0.8780	0.8939	0.8923	0.9604	0.8694
Fold average	0.7958	0.8128	0.8863	0.8119	0.8694	0.8745	0.8680	0.9505	**0.8586**

Bold values ​​are the averages across all folds for all subjects.

Table 2 reports the performance of a single pooled P300MCNN trained using data from all eight subjects. Unlike SQ_EMoE, this baseline does not perform subject-dependent expert routing and therefore provides an architecture-related reference for assessing whether the performance gain of SQ_EMoE originates from the proposed routing strategy rather than solely from the underlying P300 decoder. The pooled P300MCNN achieved an overall mean ROC–AUC of 0.8317, compared with 0.8586 for SQ_EMoE. The approximately 0.03 absolute improvement obtained by SQ_EMoE suggests that its performance advantage cannot be attributed solely to the use of the lightweight P300 classifier architecture.

**Table 2:** Subject-wise ROC–AUC of the pooled P300MCNN baseline.

Folds	Subjects								
	Subject 0	Subject 1	Subject 2	Subject 3	Subject 4	Subject 5	Subject 6	Subject 7	Subject mean
Fold 0	0.7945	0.7819	0.8558	0.8164	0.8107	0.8298	0.8172	0.9037	0.8262
Fold 1	0.8296	0.8168	0.8756	0.7629	0.8272	0.8183	0.8603	0.9096	0.8375
Fold 2	0.7917	0.7764	0.8431	0.7478	0.8349	0.8472	0.8397	0.9264	0.8259
Fold 3	0.7638	0.7795	0.8892	0.8208	0.8389	0.7925	0.8322	0.9254	0.8303
Fold 4	0.8129	0.7754	0.8966	0.7992	0.8288	0.8173	0.8646	0.9157	0.8388
Fold average	0.7985	0.7860	0.8721	0.7894	0.8281	0.8210	0.8428	0.9162	**0.8317**

Bold values ​​are the averages across all folds for all subjects.

Table 3 summarizes the performance of the pooled EEGNet baseline under the same eight-subject evaluation setting. EEGNet achieved an overall mean ROC–AUC of 0.8323 close to P300MCNN baseline, which was lower than the 0.8586 achieved by SQ_EMoE. The result is broadly consistent with that obtained using the pooled P300MCNN baseline and provides an additional architecture-independent reference showing that the complete SQ_EMoE framework achieves higher overall decoding performance than conventional pooled cross-subject models due to its overall advantages.

**Table 3:** Subject-wise ROC–AUC of the pooled EEGNet baseline.

Folds	Subjects								
	Subject 0	Subject 1	Subject 2	Subject 3	Subject 4	Subject 5	Subject 6	Subject 7	Subject mean
Fold 0	0.7950	0.7889	0.8300	0.8154	0.8451	0.8161	0.8338	0.8970	0.8277
Fold 1	0.8266	0.8155	0.8694	0.7667	0.8445	0.8270	0.8558	0.8971	0.8378
Fold 2	0.7899	0.7726	0.8326	0.7562	0.8487	0.8412	0.8544	0.9134	0.8261
Fold 3	0.7656	0.7726	0.8754	0.8118	0.8540	0.7916	0.8606	0.9293	0.8326
Fold 4	0.8118	0.8006	0.8838	0.7955	0.8332	0.7994	0.8693	0.9057	0.8374
Fold average	0.7978	0.7900	0.8582	0.7891	0.8451	0.8151	0.8548	0.9085	**0.8323**

Bold values ​​are the averages across all folds for all subjects.

Table 4 evaluates an ablated version of SQ_EMoE in which the general expert and confidence-based fallback mechanism are removed. In this configuration, each trial is directly assigned to the subject-specific expert selected by the subject-identification network, irrespective of routing confidence. The resulting model achieved a mean ROC–AUC of 0.8468, compared with approximately 0.8586 for the complete SQ_EMoE framework. The performance reduction after removing the general expert supports the role of confidence-based fallback in handling trials for which subject-specific routing is uncertain. Nevertheless, the relatively strong performance of the expert-only configuration also indicates that subject-specific expert specialization itself constitutes an important component of the overall framework.

**Table 4:** Ablation study of SQ_EMoE without the general expert.

Folds	Subjects								
	Subject 0	Subject 1	Subject 2	Subject 3	Subject 4	Subject 5	Subject 6	Subject 7	Subject mean
Fold 0	0.7608	0.7824	0.8842	0.8325	0.8384	0.8754	0.8512	0.9206	0.8432
Fold 1	0.7940	0.7556	0.8951	0.7896	0.8089	0.8725	0.8614	0.9582	0.8419
Fold 2	0.7821	0.8256	0.8563	0.7938	0.8022	0.8815	0.8458	0.9488	0.8420
Fold 3	0.8051	0.7953	0.8771	0.8283	0.8642	0.8655	0.8043	0.9560	0.8495
Fold 4	0.7922	0.8211	0.8746	0.8131	0.8615	0.8798	0.8747	0.9443	0.8577
Fold average	0.7868	0.7960	0.8774	0.8114	0.8350	0.8749	0.8475	0.9456	**0.8468**

Bold values ​​are the averages across all folds for all subjects.

#### 3.1.2 P300-LINI

As shown in Figure 4, the right panel illustrates the 2D t-SNE projection of the network’s 64-dimensional subject embedding layer. Very different from Figure 3, the latent feature space displays more complex topological characteristics. While data points from the same subject still aggregate into localized dense regions, there is visible proximity and minor cluster cross-contamination along the boundaries of adjacent subject clusters. This spatial overlapping directly mirrors the high routing error rate reported in the left panel, visually representing the low subject variability and shared spatial–temporal patterns that make subject identification exceptionally difficult. In this case, more samples will be routed to the general experts.

**Figure 4:**
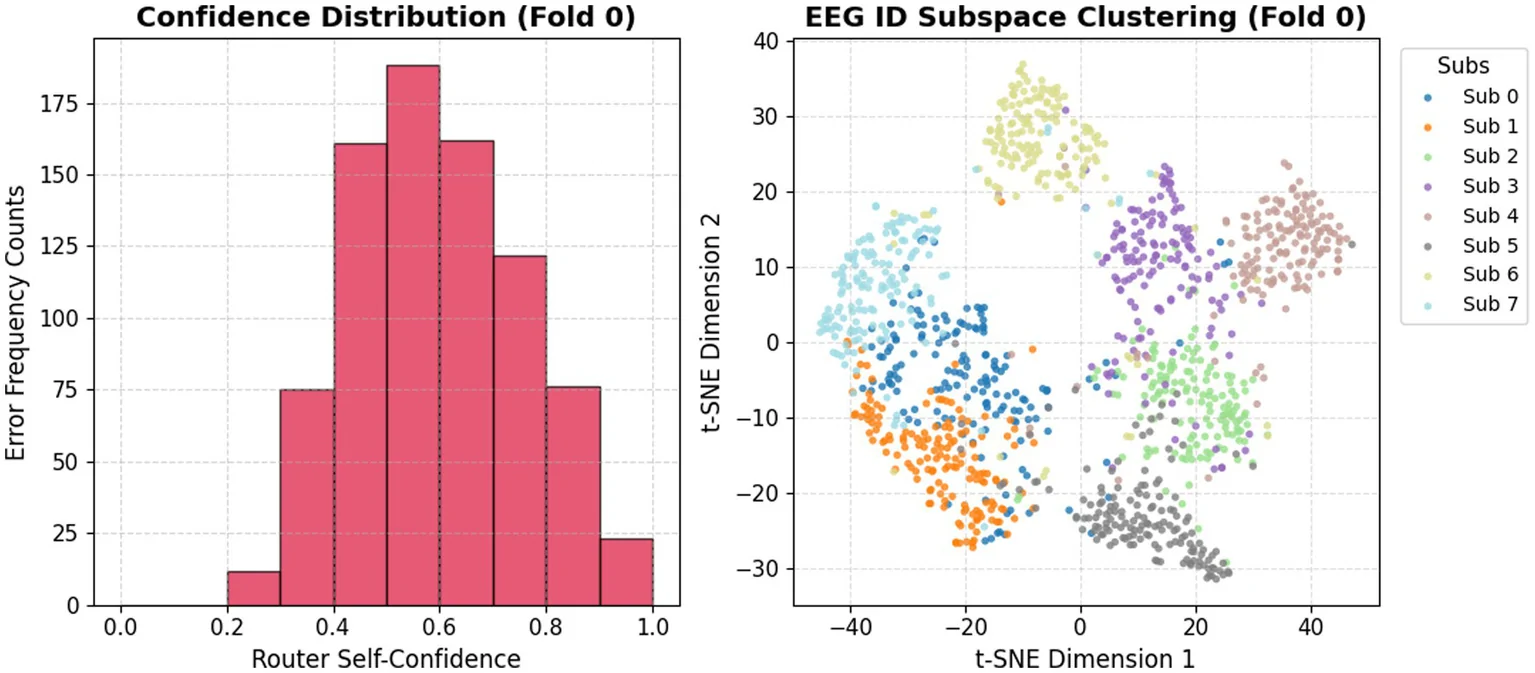
Subject identification and routing analysis for eight subjects from the P300-LINI dataset. (Left) Distribution of router confidence scores for incorrect routing instances. (Right) Two-dimensional t-SNE visualization of the 64-dimensional subject embeddings learned by the subject identification network.

The left panel plots the network’s prediction confidence levels specifically for the trials that were incorrectly routed. Compared to the previous Figure 3, the distribution curve reveals a sharp decline, with error points heavily concentrated in low-confidence zones. Nevertheless, by adjusting the threshold, the routing unit can effectively flag these low-confidence instances in real time, redirecting them from specialized sub-experts to the general expert to safeguard system stability.

Table 5 reports the five-fold cross-validation performance of SQ_EMoE on eight randomly selected subjects from the P300-LINI dataset. The framework achieved an overall mean ROC–AUC of 0.8908, with subject-wise averages ranging from approximately 0.87 to 0.92. The relatively consistent fold-wise performance indicates that the proposed framework remains stable across different train-test partitions. Together with the Horizon results, these findings demonstrate that SQ_EMoE can be applied across datasets exhibiting different subject-dependent decoding characteristics.

**Table 5:** Subject-wise P300 decoding performance of SQ_EMoE across eight random subjects.

Folds	Subjects								
	Subject 0	Subject 1	Subject 5	Subject 8	Subject 11	Subject 13	Subject 15	Subject 20	Subject mean
Fold 0	0.8779	0.9310	0.9233	0.8753	0.8492	0.9050	0.9080	0.8858	0.8944
Fold 1	0.9046	0.8875	0.9457	0.8879	0.8791	0.8655	0.8749	0.9419	0.8984
Fold 2	0.8589	0.8243	0.9033	0.8638	0.8650	0.8897	0.9060	0.9377	0.8811
Fold 3	0.8617	0.9033	0.8990	0.8539	0.8757	0.8932	0.8794	0.8992	0.8832
Fold 4	0.8869	0.8995	0.9128	0.8922	0.8761	0.9040	0.9021	0.9018	0.8969
Fold average	0.8780	0.8891	0.9168	0.8746	0.8690	0.8915	0.8941	0.9133	**0.8908**

Bold values ​​are the averages across all folds for all subjects.

Table 6 presents the performance of the pooled P300MCNN baseline on the same eight P300-LINI subjects. The model achieved an overall mean ROC–AUC of 0.8755, compared with 0.8908 for SQ_EMoE. Because the pooled baseline uses the same underlying lightweight decoding architecture without subject-dependent expert routing, this comparison provides a more controlled assessment of the contribution of the SQ_EMoE routing mechanism. The improvement over the pooled P300MCNN is smaller than that observed on the Horizon dataset, suggesting that, the smaller the subject variability is in the dataset, the better the pooled model baseline performs; conversely, the greater the subject variability is in the dataset, the better the MoE model performs. SQ_EMoE can balance the two by adjusting the confidence threshold.

**Table 6:** Subject-wise ROC–AUC of the pooled P300MCNN baseline.

Folds	Subjects								
	Subject 0	Subject 1	Subject 5	Subject 8	Subject 11	Subject 13	Subject 15	Subject 20	Subject mean
Fold 0	0.8670	0.8478	0.8824	0.8947	0.8442	0.8756	0.8913	0.8798	0.8729
Fold 1	0.8874	0.8646	0.9105	0.8883	0.8390	0.8650	0.8346	0.9237	0.8766
Fold 2	0.8795	0.8489	0.8853	0.8711	0.8462	0.8726	0.8627	0.9227	0.8736
Fold 3	0.8845	0.8901	0.8955	0.8641	0.8600	0.8689	0.8620	0.9034	0.8786
Fold 4	0.8867	0.8553	0.9057	0.8634	0.8492	0.8814	0.8701	0.8928	0.8756
Fold average	0.8810	0.8613	0.8959	0.8763	0.8477	0.8727	0.8642	0.9045	**0.8755**

Bold values ​​are the averages across all folds for all subjects.

Table 7 reports the corresponding pooled EEGNet results on P300-LINI. EEGNet achieved an overall mean ROC–AUC of 0.8764, which is comparable to the pooled P300MCNN result of 0.8755 but lower than the approximately 0.8908 obtained with SQ_EMoE. The similar performance of the two pooled baselines is particularly informative because it suggests that the improvement observed for SQ_EMoE is unlikely to be explained simply by choosing P300MCNN rather than EEGNet as the underlying decoder.

**Table 7:** Subject-wise ROC–AUC of the pooled EEGNet baseline.

Folds	Subjects								
	Subject 0	Subject 1	Subject 5	Subject 8	Subject 11	Subject 13	Subject 15	Subject 20	Subject mean
Fold 0	0.8563	0.8736	0.8911	0.8929	0.8271	0.8834	0.8723	0.8766	0.8717
Fold 1	0.8784	0.8848	0.9159	0.8967	0.8251	0.8667	0.8337	0.9087	0.8763
Fold 2	0.8753	0.8625	0.8930	0.8636	0.8426	0.8818	0.8768	0.9237	0.8774
Fold 3	0.8770	0.8934	0.8977	0.8656	0.8487	0.8886	0.8681	0.9018	0.8801
Fold 4	0.8776	0.8639	0.8997	0.8559	0.8586	0.8848	0.8732	0.8969	0.8763
Fold average	0.8729	0.8757	0.8995	0.8749	0.8404	0.8810	0.8648	0.9015	**0.8764**

Bold values ​​are the averages across all folds for all subjects.

Table 8 examines the contribution of the general expert by forcing all trials to be processed by the subject-specific expert selected by the router. Without confidence-based fallback to the general expert, the model achieved an overall mean ROC–AUC of 0.8827. This value remains higher than those of the pooled P300MCNN (0.8755) and EEGNet (0.8764) baselines but is lower than that of the complete SQ_EMoE framework (approximately 0.8908). These results suggest that both subject-specific expert specialization and the confidence-controlled general-expert fallback contribute to the final performance, rather than the improvement being attributable to a single component.

**Table 8:** Ablation study of SQ_EMoE without the general expert.

Folds	Subjects								
	Subject 0	Subject 1	Subject 5	Subject 8	Subject 11	Subject 13	Subject 15	Subject 20	Subject mean
Fold 0	0.8227	0.9206	0.8615	0.8514	0.8479	0.8719	0.8935	0.8758	0.8682
Fold 1	0.8611	0.9138	0.9275	0.8687	0.8754	0.8457	0.8801	0.9431	0.8894
Fold 2	0.8780	0.9139	0.9048	0.8434	0.8379	0.8647	0.8646	0.9367	0.8805
Fold 3	0.8502	0.9070	0.8940	0.8581	0.8391	0.8895	0.8961	0.9118	0.8807
Fold 4	0.8806	0.9166	0.9313	0.8697	0.8816	0.8864	0.8859	0.9045	0.8946
Fold average	0.8585	0.9144	0.9038	0.8583	0.8564	0.8717	0.8840	0.9144	**0.8827**

Bold values ​​are the averages across all folds for all subjects.

### 3.2 Unseen-subject evaluation

While the known-subject setting represents the primary evaluation paradigm for the proposed framework, its performance under unseen-subject scenarios remains a key consideration. In our LOSO experiments on unseen subjects, approximately 99% of trials are routed to the integrated general expert, resulting in performance that is comparable to, though only modestly higher than, the pooled baseline. When encountering unseen data, the framework effectively falls back to its general-purpose model by adjusting the confidence threshold, ensuring robust baseline performance. Expanding the expert pool with many more subjects could potentially enhance model selection for unseen individuals, narrowing the gap between unseen- and known-subject performance. However, validating this hypothesis remains an avenue for future research due to the limited subject pools in current benchmark datasets.

In the unseen-subject setting, LOSO evaluation was performed on both datasets, with each test subject completely excluded from model training. These experiments assess the generalization of SQ_EMoE to previously unseen subjects and examine how the general expert serves as a fallback when no subject-specific expert is available for the test subject.

#### 3.2.1 BNCI horizon 2020

Table 9 evaluates SQ_EMoE under a LOSO protocol to investigate its generalization to completely unseen subjects. In each experiment, one subject was entirely excluded from training and subsequently used only for testing. SQ_EMoE achieved a mean ROC–AUC of 0.7819 across the eight held-out subjects. Importantly, approximately 99.8% of the unseen-subject trials were routed to the general expert under the high-confidence routing criterion. This behavior indicates that the routing mechanism generally refrains from assigning unseen-subject samples to potentially inappropriate subject-specific experts when sufficient routing confidence is unavailable. Instead, the pooled general expert serves as the dominant fallback pathway for previously unseen users.

**Table 9:** Leave-one-subject-out evaluation of SQ_EMoE on unseen.

Test results	Subjects held out								
	Subject 0	Subject 1	Subject 2	Subject 3	Subject 4	Subject 5	Subject 6	Subject 7	Average
ROC–AUC	0.7634	0.7552	0.8385	0.7410	0.7524	0.7784	0.7867	0.8395	**0.7819**
Routing results	E5: 2	General: 4,200	E5: 1	General: 4,200	General: 4,200	General: 4,200	E1: 49	General: 4,200	99.8% routed to the general model
	E6:1		E6:18						
			General: 4,181						
							General: 4,151		
	General: 4,197								

Bold values ​​are the averages across all folds for all subjects.

Table 10 reports the LOSO performance of the pooled EEGNet baseline on the BNCI Horizon dataset. For each LOSO iteration, EEGNet was trained using data from all available training subjects and evaluated exclusively on the held-out subject. The model achieved a mean ROC–AUC of 0.7809 across the eight unseen subjects, close but lower than SQ_EMoE. The gap is very small because only 0.2% were routed to subject-specific experts in SQ_EMoE and statistical evaluations shows that this improvement is not statistically significant. In this case, SQ_EMoE functions closer to a general model due to the limited subject pool. We hypothesize that SQ_EMoE’s design philosophy—relying on a massive expert pool—operates on a different premise than traditional LOSO evaluation, a hypothesis we plan to validate in future work.

**Table 10:** Leave-one-subject-out performance of the pooled EEGNet.

Test results	Subjects held out								
	Subject 0	Subject 1	Subject 2	Subject 3	Subject 4	Subject 5	Subject 6	Subject 7	Average
ROC–AUC	0.7620	0.7581	0.8344	0.7419	0.7624	0.7733	0.7818	0.8332	**0.7809**

Bold values ​​are the averages across all folds for all subjects.

#### 3.2.2 P300-LINI

Table 11 further evaluates unseen-subject generalization using LOSO on the P300-LINI dataset. Across the eight held-out subjects, SQ_EMoE achieved a mean ROC–AUC of 0.8499. Under the conservative routing threshold, approximately 99.1% of the held-out trials were directed to the general expert, while only a small fraction was assigned to one of the known subject-specific experts. This result is consistent with the Horizon LOSO experiment and illustrates the intended fallback behavior of SQ_EMoE for users who were not represented during training: rather than requiring an existing subject-specific expert for the new user, the framework predominantly relies on the pooled general expert when the subject router does not provide sufficiently strong evidence for a known identity. We hypothesize that fully configuring SQ_EMoE with a substantial number of diverse experts could help improve this situation in future work.

**Table 11:** Leave-one-subject-out evaluation of SQ_EMoE on unseen subjects.

Test results	Subjects held out								
	Subject 0	Subject 1	Subject 5	Subject 8	Subject 11	Subject 13	Subject 15	Subject 20	Average
ROC–AUC	0.8811	0.8251	0.8851	0.8565	0.8004	0.8564	0.8056	0.8892	**0.8499**
Routing results	General: 2,880	General: 2,880	E13: 69	E5: 74	General: 2,880	E1: 1	E8: 8	E8: 16	99.1% routed to the general model
						E5: 16			
				E15: 21					
						General: 2,863	General: 2,872	General: 2,864	
			General: 2,811	E20: 3					
				General: 2,782					

Bold values ​​are the averages across all folds for all subjects.

Table 12 presents the corresponding LOSO results of the pooled EEGNet baseline on the P300-LINI dataset. Across the eight held-out subjects, EEGNet achieved a mean ROC–AUC of *0.8467*. Similar to the Horizon experiment, this result serves as a conventional pooled cross-subject reference for evaluating the behavior of SQ_EMoE when the test subject is completely absent from training.

**Table 12:** Leave-one-subject-out performance of the pooled EEGNet.

Test results	Subjects held out								
	Subject 0	Subject 1	Subject 5	Subject 8	Subject 11	Subject 13	Subject 15	Subject 20	Average
ROC–AUC	0.8754	0.8242	0.8846	0.8619	0.7846	0.8639	0.7952	0.8836	**0.8467**

Bold values ​​are the averages across all folds for all subjects.

The LOSO test of SQ_EMoE performs better on the P300-LINI dataset because the subject variability in P300-LINI is lower than in BNCI Horizon, resulting in a higher probability of finding a relatively well-matched expert model for unseen subjects.

### 3.3 Known-subject evaluation across 22 subjects

The evaluations discussed above—covering both known and unseen subjects—were based on a set of eight random subjects. The subject identification network used in those evaluations was too simplistic to effectively handle the task of identifying a larger number of subjects. As illustrated in Figure 5, thousands of errors occur as the majority of the 22 subjects overlap within the subspace clustering, severely hindering subject separability. Consequently, as presented in Table 13, the ROC–AUC reaches only 0.8518—a performance slightly higher than the general model baseline of 0.8482 in Table 14. To fully unlock SQ_EMoE’s potential, we upgraded the original identification network to accommodate 22 subjects and compared its performance against the previous version to complete ablation study. Additionally, we evaluated performance across various confidence thresholds and compared the results with oracle routing as well.

**Figure 5:**
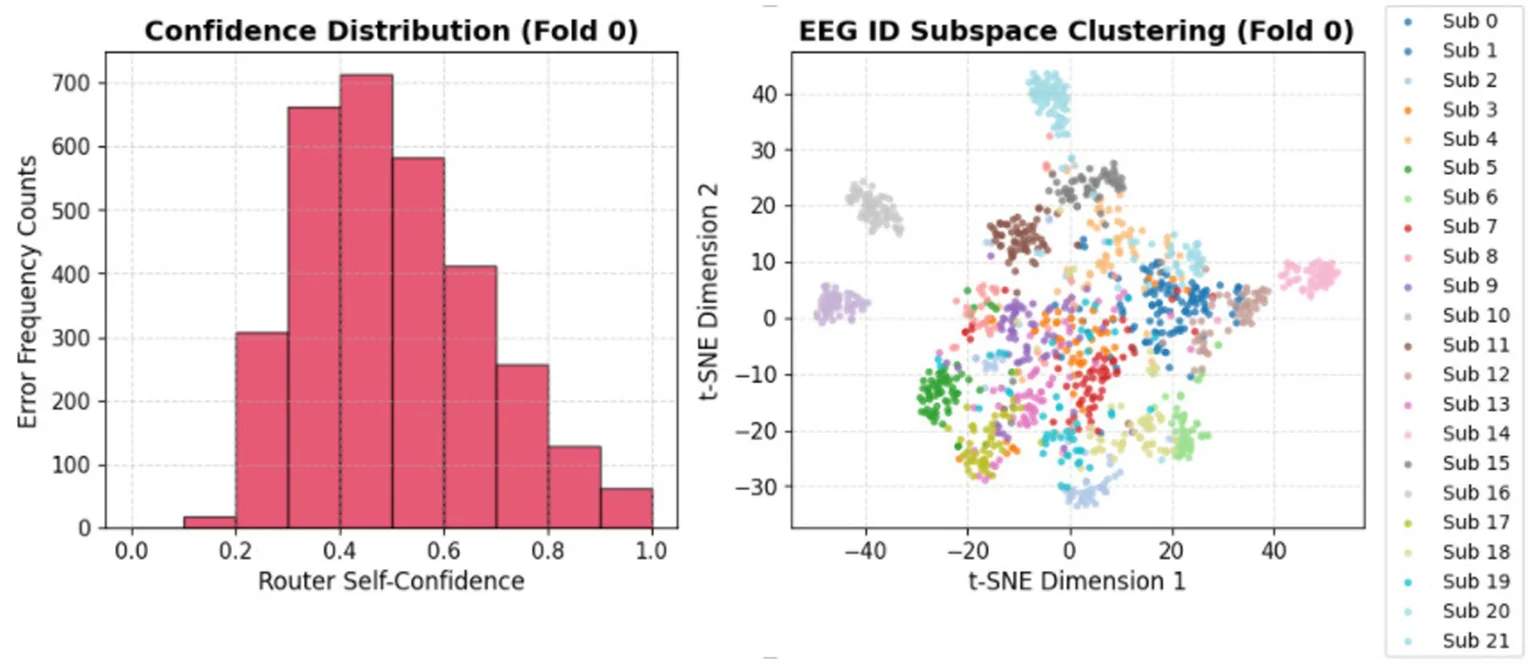
Subject identification and routing analysis for all 22 subjects from the P300-LINI dataset using the original subject identification network. (Left) Distribution of router confidence scores for incorrect routing instances. (Right) Two-dimensional t-SNE visualization of the learned subject embeddings.

**Table 13:** Subject-wise ROC–AUC of SQ_EMoE across 22 subjects using the original subject identification network with the confidence threshold of 0.7.

Folds	Subjects										
	Subject 0	Subject 1	Subject 2	Subject 3	Subject 4	Subject 5	Subject 6	Subject 7	Subject 8	Subject 9	Subject 10
Fold 0	0.8383	0.9099	0.8943	0.6529	0.6971	0.8904	0.7718	0.8155	0.9085	0.8661	0.8697
Fold 1	0.8886	0.8729	0.8784	0.7041	0.7266	0.9034	0.7664	0.8128	0.8963	0.8519	0.7574
Fold 2	0.8717	0.8871	0.8847	0.7503	0.7211	0.8930	0.7678	0.8272	0.8842	0.8610	0.8537
Fold 3	0.8785	0.9275	0.9153	0.7954	0.7346	0.9075	0.7827	0.8303	0.8604	0.8756	0.8561
Fold 4	0.8781	0.9046	0.8657	0.7281	0.7175	0.9129	0.8132	0.7807	0.8424	0.8541	0.7261
Fold average	0.8710	0.9004	0.8877	0.7262	0.7194	0.9014	0.7804	0.8133	0.8784	0.8617	0.8126

Folds	Subjects											
	Subject 11	Subject 12	Subject 13	Subject 14	Subject 15	Subject 16	Subject 17	Subject 18	Subject 19	Subject 20	Subject 21	Subject mean
Fold 0	0.8677	0.8499	0.8875	0.8663	0.8983	0.8198	0.8999	0.9231	0.8692	0.8837	0.8375	0.8508
Fold 1	0.8439	0.8247	0.8718	0.8601	0.8485	0.7787	0.9154	0.9182	0.9193	0.9374	0.8660	0.8474
Fold 2	0.8581	0.8284	0.8649	0.7067	0.8929	0.7965	0.9200	0.9071	0.9201	0.9336	0.8727	0.8501
Fold 3	0.8685	0.8091	0.8635	0.8451	0.8999	0.8327	0.9070	0.9352	0.9242	0.9225	0.8275	0.8636
Fold 4	0.8764	0.8203	0.8794	0.8598	0.8853	0.8037	0.9463	0.9132	0.8839	0.9064	0.8387	0.8471
Fold average	0.8629	0.8265	0.8734	0.8276	0.8850	0.8063	0.9177	0.9194	0.9033	0.9167	0.8485	**0.8518**

Bold values ​​are the averages across all folds for all subjects.

**Table 14:** Subject-wise ROC–AUC of the pooled P300MCNN baseline.

Folds	Subjects										
	Subject 0	Subject 1	Subject 2	Subject 3	Subject 4	Subject 5	Subject 6	Subject 7	Subject 8	Subject 9	Subject 10
Fold 0	0.8450	0.8736	0.9073	0.6788	0.7252	0.8651	0.7775	0.7967	0.9058	0.8644	0.8364
Fold 1	0.8730	0.8751	0.9011	0.7222	0.6945	0.8990	0.7586	0.8015	0.9014	0.8567	0.8275
Fold 2	0.8637	0.8596	0.9153	0.7724	0.7238	0.8792	0.7861	0.8389	0.8734	0.8850	0.8412
Fold 3	0.8637	0.8854	0.9162	0.7552	0.7417	0.8840	0.8128	0.8111	0.8576	0.8781	0.8644
Fold 4	0.8704	0.8464	0.9084	0.7376	0.7260	0.8928	0.8200	0.8287	0.8506	0.8587	0.8152
Fold average	0.8631	0.8680	0.9097	0.7332	0.7222	0.8840	0.7910	0.8154	0.8777	0.8686	0.8369

Folds	Subjects											
	Subject 11	Subject 12	Subject 13	Subject 14	Subject 15	Subject 16	Subject 17	Subject 18	Subject 19	Subject 20	Subject 21	Subject mean
Fold 0	0.8007	0.8372	0.8695	0.8501	0.8929	0.7496	0.8953	0.9459	0.8611	0.8965	0.8646	0.8427
Fold 1	0.8195	0.8112	0.8666	0.8713	0.8500	0.6950	0.8893	0.9298	0.9047	0.9292	0.8841	0.8437
Fold 2	0.8362	0.8340	0.8755	0.8666	0.8656	0.7675	0.9138	0.9223	0.9221	0.9231	0.8605	0.8557
Fold 3	0.8411	0.8589	0.8814	0.8119	0.8595	0.7569	0.9038	0.9485	0.9091	0.9137	0.8071	0.8528
Fold 4	0.8250	0.8237	0.8847	0.8513	0.8814	0.7308	0.9186	0.9357	0.8964	0.8912	0.8164	0.8459
Fold average	0.8245	0.8330	0.8755	0.8502	0.8699	0.7400	0.9042	0.9364	0.8987	0.9107	0.8465	**0.8482**

Bold values ​​are the averages across all folds for all subjects.

For a more comprehensive comparison, the results for oracle routing are also presented; as shown in Table 15, the final ROC–AUC for oracle routing stands at 0.8744, much higher than 0.8518 of the previous SQ_EMoE with original subject identification network and 0.8482 of the pooled P300MCNN baseline. Oracle routing refers to a scenario where all routing decisions are 100% correct, analogous to within-subject decoding. The following presents the performance of SQ_EMoE with the upgraded subject identification network and a comparison with oracle routing.

**Table 15:** Subject-wise ROC–AUC of oracle routing of 22 subjects.

Folds	Subjects										
	Subject 0	Subject 1	Subject 2	Subject 3	Subject 4	Subject 5	Subject 6	Subject 7	Subject 8	Subject 9	Subject 10
Fold 0	0.8935	0.9389	0.8977	0.6416	0.7910	0.9329	0.7812	0.8472	0.9213	0.8994	0.8763
Fold 1	0.9308	0.9041	0.9121	0.7199	0.8240	0.9494	0.7669	0.8199	0.9113	0.8895	0.7626
Fold 2	0.9081	0.9109	0.9025	0.7485	0.8423	0.9425	0.7399	0.8628	0.8872	0.9077	0.8604
Fold 3	0.9006	0.9337	0.9282	0.7683	0.8334	0.9354	0.8265	0.8311	0.8725	0.8835	0.8226
Fold 4	0.9029	0.9068	0.8837	0.7242	0.8127	0.9423	0.8077	0.8331	0.9045	0.9077	0.8599
Fold average	0.9072	0.9189	0.9048	0.7205	0.8207	0.9405	0.7845	0.8388	0.8993	0.8976	0.8364

Folds	Subjects											
	Subject 11	Subject 12	Subject 13	Subject 14	Subject 15	Subject 16	Subject 17	Subject 18	Subject 19	Subject 20	Subject 21	Subject mean
Fold 0	0.8659	0.8712	0.9156	0.8694	0.9135	0.8187	0.9602	0.9485	0.9090	0.9146	0.8361	0.8747
Fold 1	0.8642	0.8169	0.8653	0.8477	0.9071	0.7430	0.9764	0.9280	0.9421	0.9449	0.8448	0.8668
Fold 2	0.8847	0.8480	0.8920	0.8269	0.9143	0.8395	0.9530	0.9293	0.9552	0.9364	0.8597	0.8796
Fold 3	0.8616	0.8533	0.8972	0.8428	0.9077	0.8525	0.9588	0.9321	0.9338	0.9039	0.8241	0.8774
Fold 4	0.8923	0.8416	0.8936	0.8262	0.9079	0.7960	0.9674	0.9394	0.9361	0.9007	0.8253	0.8733
Fold average	0.8737	0.8462	0.8927	0.8426	0.9101	0.8099	0.9631	0.9355	0.9352	0.9201	0.8380	**0.8744**

Bold values ​​are the averages across all folds for all subjects.

Figure 6 illustrates the qualitative performance of the upgraded subject identification network. The updated architecture significantly improves subject discriminability, yielding distinct clusters for the 22 subjects. While minor classification errors remain, this represents a stark contrast to the severe cluster overlap observed in Figure 5. Correspondingly, decoding errors plummeted from several thousand to merely a few hundred, driving a substantial boost in ROC–AUC. As reported in Table 16, the final ROC–AUC reached 0.8760, outperforming both the original version (0.8518) and the oracle routing (0.8744).

**Figure 6:**
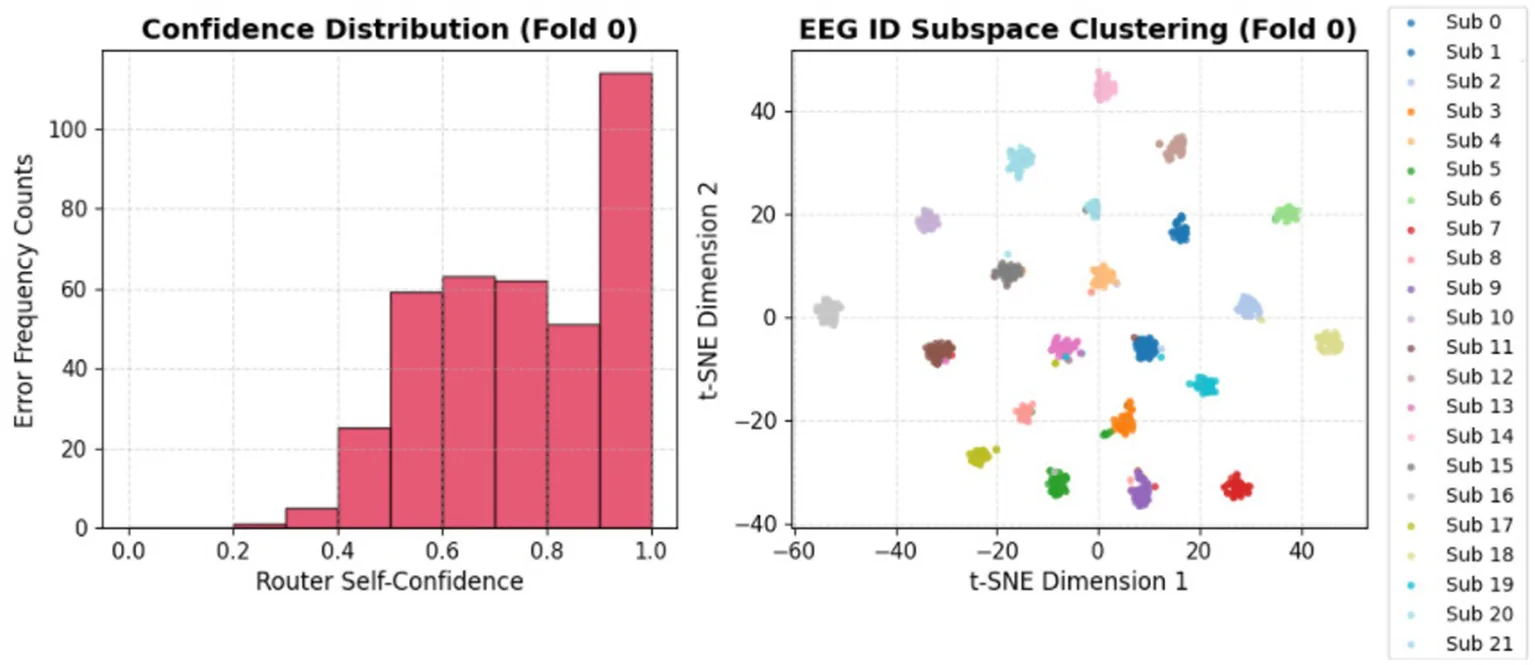
Subject identification and routing analysis for all 22 subjects from the P300-LINI dataset using the upgraded subject identification network. (Left) Distribution of router confidence scores for incorrect routing instances. (Right) Two-dimensional t-SNE visualization of the learned subject embeddings.

**Table 16:** Subject-wise ROC–AUC of SQ_EMoE across 22 subjects using the upgraded subject identification network with the confidence threshold of 0.7.

Folds	Subjects										
	Subject 0	Subject 1	Subject 2	Subject 3	Subject 4	Subject 5	Subject 6	Subject 7	Subject 8	Subject 9	Subject 10
Fold 0	0.8941	0.9205	0.9060	0.6464	0.7833	0.9325	0.7923	0.8310	0.9172	0.9037	0.8642
Fold 1	0.9171	0.9050	0.9071	0.7098	0.8000	0.9392	0.7849	0.8138	0.9153	0.8972	0.8747
Fold 2	0.9003	0.9322	0.9020	0.7547	0.8733	0.9255	0.7642	0.8636	0.9120	0.8839	0.8526
Fold 3	0.8729	0.9432	0.9228	0.7850	0.8347	0.9339	0.8122	0.8308	0.8934	0.8955	0.8722
Fold 4	0.8853	0.9017	0.8860	0.7292	0.7923	0.9376	0.8114	0.8401	0.8982	0.9057	0.8474
Fold average	0.8939	0.9205	0.9048	0.7250	0.8167	0.9337	0.7930	0.8358	0.9072	0.8972	0.8622

Folds	Subjects											
	Subject 11	Subject 12	Subject 13	Subject 14	Subject 15	Subject 16	Subject 17	Subject 18	Subject 19	Subject 20	Subject 21	Subject mean
Fold 0	0.8690	0.8458	0.9083	0.8806	0.9013	0.8213	0.9527	0.9346	0.8981	0.9069	0.8393	0.8704
Fold 1	0.8589	0.8070	0.8677	0.8557	0.9017	0.8062	0.9707	0.9232	0.9342	0.9497	0.8864	0.8739
Fold 2	0.8747	0.8359	0.8749	0.8490	0.9167	0.8360	0.9602	0.9306	0.9497	0.9351	0.8461	0.8806
Fold 3	0.8732	0.8298	0.8996	0.8647	0.9339	0.8463	0.9590	0.9412	0.9355	0.9058	0.8283	0.8824
Fold 4	0.8874	0.8422	0.8921	0.8627	0.9087	0.7896	0.9605	0.9419	0.9364	0.9114	0.8336	0.8728
Fold average	0.8726	0.8321	0.8885	0.8625	0.9124	0.8199	0.9606	0.9343	0.9308	0.9218	0.8467	**0.8760**

Bold values ​​are the averages across all folds for all subjects.

To rigorously validate model performance, we conducted paired statistical evaluations across all 22 subjects, reporting subject-level *p*-values, Cohen’s *d* effect sizes, and 95% bootstrap confidence intervals (CIs) for performance differences. For each subject, the primary performance metric (ROC–AUC) was defined as the average performance across all 5 cross-validation folds. Differences between proposed and baseline models were evaluated using two-tailed Wilcoxon signed-rank tests (non-parametric paired tests). The magnitude of improvement was quantified using paired Cohen’s d , computed as $d=\bar{D}/s_{D}$ , where $\bar{D}$ and $s_{D}$ represent the sample mean and standard deviation of the paired subject-level AUC differences ( $D_{i}=\mathrm{AU}C_{\text{upgraded},i}-\mathrm{AU}C_{\text{baseline},i}$ ), respectively. To estimate the precision of performance gains, 95% CIs for the mean difference were constructed using a percentile bootstrap method with 10,000 resamples drawn with replacement at the subject level. Statistical significance was declared at *p* < 0.05.

The upgraded model achieves an overall mean ROC–AUC of 0.8760. Compared with the original model (0.8518) and the general model (0.8482), the upgraded model provides statistically significant improvements of +0.0242 (*p* < 0.001, Cohen’s *d* = 1.143) and +0.0279 (*p* < 0.001, Cohen’s *d* = 1.008), respectively. Their 95% CIs for AUC differences strictly exclude zero [(+0.0164, +0.0338) and (+0.0171, +0.0394)], confirming large and robust effect sizes across subjects. While the upgraded model shows a minor numerical margin over the oracle routing model (+0.0016 AUC), this difference is not statistically significant (*p* = 0.6143, Cohen’s *d* = 0.175).

We further evaluated the upgraded model’s performance across various confidence thresholds. As summarized in Table 17, the ROC–AUC exhibits a parabolic trend as the threshold increases from 0.6 to 0.8, initially rising before declining and peaking at a threshold of 0.7, with the result of 0.8760.

**Table 17:** ROC–AUC scanning different confidence thresholds.

Confidence thresholds	0.6	0.65	0.675	**0.7**	0.725	0.75	0.8
ROC–AUC	0.8691	0.8738	0.8731	**0.8760**	0.8736	0.8703	0.8667

Bold values ​​are the averages across all folds for all subjects.

## 4 Conclusion

This study introduced SQ_EMoE, a subject-variability-aware Mixture-of-Experts framework for cross-subject binary P300 decoding. Instead of assuming that either a single generalized decoder or fully subject-specific models are universally optimal, SQ_EMoE uses the confidence produced by a subject-identification network as a practical trial-level proxy for subject distinctiveness. Trials that can be confidently associated with a known subject are routed to the corresponding subject-specific expert, whereas uncertain trials are redirected to a pooled general expert. The framework therefore dynamically balances subject-specific specialization with the exploitation of representations shared across subjects.

Five-fold cross-validation experiments on eight subjects from each of the BNCI Horizon 2020 and P300-LINI datasets yielded mean ROC–AUC values of approximately 0.86 and 0.89, respectively. SQ_EMoE consistently outperformed both pooled EEGNet and architecture-matched pooled P300MCNN baselines. Ablation experiments further showed that forcing all trials through subject-specific experts reduced performance, supporting the contribution of confidence-controlled fallback to the general expert in addition to expert specialization itself. The subject-embedding visualizations provided complementary qualitative evidence that the learned subject representation is closely associated with routing behavior: greater subject separability was accompanied by more reliable subject-specific routing, whereas overlapping representations resulted in greater routing uncertainty and increased reliance on the general expert. These observations support the central motivation of SQ_EMoE that the relative contributions of shared and subject-specific information should be handled adaptively rather than through a fixed decoding strategy.

Importantly, the additional 22-subject P300-LINI experiment further clarified the role of the subject-identification component in the overall framework. When the original identification network was directly scaled from eight to 22 subjects, substantial overlap appeared in the learned subject space and SQ_EMoE achieved a mean ROC–AUC of only 0.8518, only slightly above the pooled P300MCNN baseline of 0.8482. After strengthening the subject representation and identification network, subject separability improved substantially and the mean ROC–AUC increased to 0.8760. This result also slightly exceeded the oracle/within-subject reference of 0.8744. Together with the threshold analysis, which identified 0.7 as the best-performing confidence threshold among the evaluated settings, these results demonstrate that the quality of subject representation and confidence estimation is a critical determinant of effective expert routing. More importantly, the ability of SQ_EMoE to reach or slightly exceed the within-subject reference suggests that exploiting subject-specific information and shared cross-subject information need not be mutually exclusive: subject-specific experts can capture individualized characteristics, while the general expert can remain beneficial for trials whose representations are better explained by shared cross-subject structure.

The LOSO experiments provide a complementary and more conservative assessment for completely unseen subjects. SQ_EMoE achieved mean ROC–AUC values of 0.7819 on BNCI Horizon and 0.8499 on P300-LINI, compared with 0.7809 and 0.8467 for pooled EEGNet, respectively. Approximately 99.8 and 99.1% of unseen-subject trials were redirected to the general expert under the conservative confidence criterion. Thus, when no corresponding subject-specific expert exists, the routing mechanism generally avoids confidently assigning an unfamiliar user to an inappropriate known-subject expert and instead falls back to pooled cross-subject decoding. At the same time, the relatively modest improvement over the pooled baseline indicates that SQ_EMoE derives its largest benefit when useful subject-specific patterns are represented within the available expert pool.

Several limitations remain. First, Softmax confidence is an operational proxy for subject variability rather than a direct estimate of the distributional distance defined theoretically in this study, and its reliability depends on both the quality and calibration of the subject-identification network. The 22-subject experiment makes this dependency particularly evident: improving the identification representation substantially improved the downstream SQ_EMoE performance. Second, although the expanded P300-LINI experiment demonstrates scalability to 22 subjects, the present evaluation is still limited to two P300 datasets, and the expert pool remains relatively small compared with the intended scalable setting. Future work should therefore investigate better-calibrated or learnable uncertainty measures, scalable expert retrieval and selection, substantially larger and more heterogeneous subject populations, and adaptive mechanisms for previously unseen users. Extending SQ_EMoE to motor imagery, steady-state visually evoked potentials, and other physiological signals will also be necessary to determine whether confidence-guided integration of shared and subject-specific representations constitutes a general strategy for managing subject variability beyond P300 decoding.

## Data Availability

Publicly available datasets were analyzed in this study. This data can be found at: https://bnci-horizon-2020.eu/database/data-sets; https://www.researchgate.net/publication/277313125_An_Open-Access_P300_Speller_Database.

## References

[ref1] Alvarado-Gonzalez M. Fuentes-Pineda G. Cervantes-Ojeda J. (2021). A few filters are enough: convolutional neural network for P300 detection. Neurocomputing 425, 37–52. doi: 10.1016/j.neucom.2020.10.104

[ref2] Alvarado-González M. Garduño E. Bribiesca E. Yáñez-Suárez O. Medina-Bañuelos V. (2016). P300 detection based on EEG shape features. Comput. Math. Methods Med. 1:2029791. doi: 10.1155/2016/2029791 PMC4736976 26881010

[ref3] Banville H. Benchetrit Y. d’Ascoli S. Rapin J. King J.-R. (2025). *Scaling laws for decoding images from brain activity*. Available online at: https://arxiv.org/abs/2501.15322 (Accessed October 9, 2026).

[ref4] Blankertz B. Lemm S. Treder M. Haufe S. Müller K. R. (2011). Single-trial analysis and classification of ERP components—a tutorial. NeuroImage 56, 814–825. doi: 10.1016/j.neuroimage.2010.06.048 20600976

[ref5] Brunner C. Müller-Putz G. (2014). BNCI Horizon—The Future of Brain/Neural Computer Interaction: Horizon 2020. Graz University of Technology. doi: 10.3217/978-3-85125-379-5

[ref6] Cecotti H. Graser A. (2010). Convolutional neural networks for P300 detection with application to brain-computer interfaces. IEEE Trans. Pattern Anal. Mach. Intell. 33, 433–445. doi: 10.1109/tpami.2010.125 20567055

[ref7] Ding J.-E. Zilverstand A. Yang S. Yang A. C.-C. Liu F. (2025). *Variational mixture of graph neural experts for Alzheimer’s disease biomarker recognition in EEG brain networks*. Available online at: https://arxiv.org/abs/2510.11917 (Accessed October 9, 2026).

[ref8] Farwell L. A. Donchin E. (1988). Talking off the top of your head: toward a mental prosthesis utilizing event-related brain potentials. Electroencephalogr. Clin. Neurophysiol. 70, 510–523. doi: 10.1016/0013-4694(88)90149-6, 2461285

[ref9] Fazel-Rezai R. Allison B. Z. Guger C. Sellers E. W. Kleih S. C. Kübler A. (2012). P300 brain computer interface: current challenges and emerging trends. Front. Neuroeng. 5:14. doi: 10.3389/fneng.2012.00014, 22822397 PMC3398470

[ref10] Fedus W. Zoph B. Shazeer N. (2022). *Switch transformers: scaling to trillion parameter models with simple and efficient sparsity*. Available online at: https://arxiv.org/abs/2101.03961 (Accessed October 9, 2026).

[ref11] Gui Y. Chen M. Su Y. Luo G. Yang Y. (2024). *EEGMamba: bidirectional state space model with mixture of experts for EEG multi-task classification*. Available online at: https://arxiv.org/abs/2407.20254 (Accessed October 9, 2026).

[ref12] Hajinoroozi M. Mao Z. Jung T.-P. Lin C.-T. Huang Y. (2016). EEG-based prediction of driver’s cognitive performance by deep convolutional neural network. Signal Process. Image Commun. 47, 549–555. doi: 10.1016/j.image.2016.05.018

[ref13] Hoffmann J. Borgeaud S. Mensch A. Buchatskaya E. Cai T. Rutherford E. . (2022). Training compute-optimal large language models. arXiv (Preprint. arXiv:2203.15556) 10. doi: 10.48550/arXiv.2203.15556

[ref14] Jacobs R. A. Jordan M. I. Nowlan S. J. Hinton G. E. (1991). Adaptive mixtures of local experts. Neural Comput. 3, 79–87. doi: 10.1162/neco.1991.3.1.79 31141872

[ref15] Jiang A. Q. Sablayrolles A. Roux A. Mensch A. Savary B. Bamford C. . (2024) *Mixtral of experts*. Available online at: https://arxiv.org/abs/2401.04088 (Accessed October 9, 2026).

[ref16] Jiang W.-B. Zhao L. Lu B.-L. Large brain model for learning generic representations with tremendous EEG data in BCI, in: International conference on learning representations, volume 2024 (2024) 16405–16426

[ref17] Klein T. Minakowski P. Sager S. (2025). Flexible patched brain transformer model for EEG decoding. Sci. Rep. 15:10935. doi: 10.1038/s41598-025-86294-3, 40157946 PMC11954987

[ref18] Kostas D. Aroca-Ouellette S. Rudzicz F. (2021). BENDR: using transformers and a contrastive self-supervised learning task to learn from massive amounts of EEG data. Front. Hum. Neurosci. 15:653659. doi: 10.3389/fnhum.2021.653659, 34248521 PMC8261053

[ref19] Kuruppu G. Wagh N. Kremen V. Varatharajah Y. (2026). EEG foundation models: a critical review of current progress and future directions. J. Neural Eng. 23:021001. doi: 10.1088/1741-2552/ae4455 41666566

[ref20] Kwon O.-Y. Lee M.-H. Guan C. Lee S.-W. (2020). Subject independent brain–computer interfaces based on deep convolutional neural networks. IEEE Trans. Neural Netw. Learn. Syst. 31, 3839–3852. doi: 10.1109/TNNLS.2019.2946869, 31725394

[ref21] Lawhern V. J. Solon A. J. Waytowich N. R. Gordon S. M. Hung C. P. Lance B. J. (2018). EEGNET: a compact convolutional neural network for EEG-based brain–computer interfaces. J. Neural Eng. 15:056013. doi: 10.1088/1741-2552/aace8c, 29932424

[ref22] Ledesma-Ramirez C. Bojorges-Valdez E. Yáñez-Suarez O. Saavedra C. Bougrain L. Gentiletti G. G. An open-access P300 speller database Fourth international brain–computer interface meeting (2010)

[ref23] Liu X.-H. Jiang W.-B. Zheng W.-L. Lu B.-L. MOGE: mixture of graph experts for cross-subject emotion recognition via decomposing EEG. In 2024 IEEE international conference on bioinformatics and biomedicine (BIBM). IEEE (2024)

[ref24] Liu M. Shi W. Zhao L. Beyette F. R. Jr. (2024). Best performance with fewest resources: unveiling the most resource-efficient convolutional neural network for P300 detection with the aid of explainable AI. Mach. Learn. Appl. 16:100542. doi: 10.1016/j.mlwa.2024.100542

[ref25] Liu M. Wang S. Beyette F. BCNN: a bantamweight convolutional neural network for P300 detection 2023 IEEE signal processing in medicine and biology symposium (SPMB) IEEE (2023) 1–6

[ref26] Liu M. Wang S. Cui W. Zhang S. Zhao L. Beyette F. R. (2026). Render EEG-based brain-computer interfaces calibration-free: trade space for time in EEG decoding. IEEE Open J. Eng. Med. Biol. 7, 94–105. doi: 10.1109/OJEMB.2026.3667029 PMC13068122 41970658

[ref27] Lotte F. Bougrain L. Cichocki A. Clerc M. Congedo M. Rakotomamonjy A. . (2018). A review of classification algorithms for EEG-based brain–computer interfaces: a 10 year update. J. Neural Eng. 15:031005. doi: 10.1088/1741-2552/aab2f2, 29488902

[ref28] Manor R. Geva A. B. (2015). Convolutional neural network for multi-category rapid serial visual presentation bci. Front. Comput. Neurosci. 9:146. doi: 10.3389/fncom.2015.00146 PMC4667102 26696875

[ref29] Riccio A. Simione L. Schettini F. Pizzimenti A. Inghilleri M. Olivetti Belardinelli M. . (2013). Attention and P300-based BCI performance in people with amyotrophic lateral sclerosis. Front. Hum. Neurosci. 7:732. doi: 10.3389/fnhum.2013.00732, 24282396 PMC3825256

[ref30] Riquelme C. Puigcerver J. Mustafa B. Neumann M. Jenatton R. Pinto A. S. . (2021). *Scaling vision with sparse mixture of experts*. Available online at: https://arxiv.org/abs/2106.05974 (Accessed October 9, 2026).

[ref31] Sellers E. W. Vaughan T. M. Wolpaw J. R. (2010). A brain-computer interface for long-term independent home use. Amyotroph. Lateral Scler. 11, 449–455. doi: 10.3109/17482961003777470, 20583947

[ref32] Shazeer N. Mirhoseini A. Maziarz K. Davis A. Le Q. Hinton G. . (2017). *Outrageously large neural networks: the sparsely-gated mixture-of-experts layer*. Available online at: https://arxiv.org/abs/1701.06538 (Accessed October 9, 2026).

[ref33] Treder M. S. Blankertz B. (2010). (C)overt attention and visual speller design in an ERP-based brain-computer interface. Behav. Brain Funct. 6, 28–13. doi: 10.1186/1744-9081-6-28, 20509913 PMC2904265

[ref34] Vaswani A. Shazeer N. Parmar N. Uszkoreit J. Jones L. Gomez A. N. . Attention is all you need, Advances in neural information processing systems 30 (2017).

[ref35] Wang X. Hersche M. Tomekce B. Kaya B. Magno M. Benini L. An accurate EEGNet-based motor imagery brain–computer interface for low-power edge computing 2020 IEEE International Symposium on Medical Measurements and Applications (MeMeA) 1–6 IEEE (2020).

[ref36] Wolpaw J. R. Brain–computer interfaces (BCIS) for communication and control Proceedings of the 9th International ACM SIGACCESS conference on computers and accessibility 1–2 (2007)

[ref37] Yang L. Sun Q. Li A. Van Hulle M. M. Are EEG foundation models worth it? Comparative evaluation with traditional decoders in diverse BCI tasks. in: The fourteenth international conference on learning representations, (2026)

[ref38] Yang C. Westover M. Sun J. BIOT: biosignal transformer for cross-data learning in the wild, Advances in Neural Information Processing Systems 36 (2023) 78240–78260

[ref39] Zander T. O. Kothe C. Jatzev S. Gaertner M. Enhancing human–computer interaction with input from active and passive brain–computer interfaces Brain–computer interfaces: applying our minds to human–computer Interaction 181–199 (2010)

[ref40] Zhai X. Kolesnikov A. Houlsby N. Beyer L. Scaling vision transformers. Proceedings of the IEEE/CVF conference on computer vision and pattern recognition (2022) 12104–12113

